# Intravenous Lidocaine for Refractory Pain in Patients With Pancreatic Ductal Adenocarcinoma and Chronic Pancreatitis: A Multicenter Prospective Nonrandomized Pilot Study

**DOI:** 10.14309/ctg.0000000000000760

**Published:** 2024-08-19

**Authors:** Simone Augustinus, Matthanja Bieze, Charlotte L. Van Veldhuisen, Marja A. Boermeester, Bert A. Bonsing, Stefan A.W. Bouwense, Marco J. Bruno, Olivier R. Busch, Werner Ten Hoope, Jan-Willem Kallewaard, Henk J. van Kranen, Marieke Niesters, Niels C.J. Schellekens, Monique A.H. Steegers, Rogier P. Voermans, Judith de Vos-Geelen, Johanna W. Wilmink, Jan H.M. Van Zundert, Casper H. van Eijck, Marc G. Besselink, Markus W. Hollmann

**Affiliations:** 1 Amsterdam UMC, University of Amsterdam, Department of Surgery, Amsterdam, the Netherlands;; 2 Cancer Center Amsterdam, Amsterdam, the Netherlands;; 3 Amsterdam UMC, University of Amsterdam, Department of Anesthesiology, Amsterdam, the Netherlands;; 4 Department of Anesthesiology and Pain Management, Toronto General Hospital, University of Toronto, Toronto, Ontario, Canada;; 5 Department of Surgery, Leiden University Medical Center, Leiden, the Netherlands;; 6 Department of Surgery, Maastricht Universitair Medisch Centrum+, Maastricht, the Netherlands;; 7 Department of Gastroenterology & Hepatology, Erasmus MC, University Medical Center, Rotterdam, the Netherlands;; 8 Department of Anesthesiology, Rijnstate Ziekenhuis, Arnhem, the Netherlands;; 9 Inspire2live, Houten, the Netherlands;; 10 Department of Anesthesiology, Leiden University Medical Center, Leiden, the Netherlands;; 11 Amsterdam UMC, University of Amsterdam, Department of Gastroenterology and Hepatology, Amsterdam, the Netherlands;; 12 Department of Medical Oncology, GROW, Maastricht University Medical Center+, Maastricht, the Netherlands;; 13 Department of Medical Oncology, Amsterdam UMC, University of Amsterdam, Amsterdam, the Netherlands;; 14 Department of Anesthesiology and Pain Medicine, Maastricht Universitair Medisch Centrum+, Maastricht, the Netherlands;; 15 Department of Anesthesiology, Intensive Care, Emergency Medicine and Multidisciplinary Pain Center, Ziekenhuis Oost-Limburg, Genk, Belgium;; 16 Department of Surgery, Erasmus MC, University Medical Center Rotterdam, Rotterdam, the Netherlands.

**Keywords:** intravenous lidocaine, chronic pancreatitis, pancreatic cancer, pain, pilot study

## Abstract

**INTRODUCTION:**

Refractory pain is a major clinical problem in patients with pancreatic ductal adenocarcinoma (PDAC) and chronic pancreatitis (CP). New, effective therapies to reduce pain are urgently needed. Intravenous lidocaine is used in clinical practice in patients with PDAC and CP, but its efficacy has not been studied prospectively.

**METHODS:**

Multicenter prospective nonrandomized pilot study included patients with moderate or severe pain (Numeric Rating Scale ≥ 4) associated with PDAC or CP in 5 Dutch centers. An intravenous lidocaine bolus of 1.5 mg/kg was followed by continuous infusion at 1.5 mg/kg/hr. The dose was raised every 15 minutes until treatment response (up to a maximum 2 mg/kg/hr) and consecutively administered for 2 hours. Primary outcome was the mean difference in pain severity, preinfusion, and the first day after (Brief Pain Inventory [BPI] scale 1–10). A BPI decrease ≥1.3 points was considered clinically relevant.

**RESULTS:**

Overall, 30 patients were included, 19 with PDAC (63%) and 11 with CP (37%). The mean difference in BPI at day 1 was 1.1 (SD ± 1.3) points for patients with PDAC and 0.5 (SD ± 1.7) for patients with CP. A clinically relevant decrease in BPI on day 1 was reported in 9 of 29 patients (31%), and this response lasted up to 1 month. No serious complications were reported, and only 3 minor complications (vertigo, nausea, and tingling of mouth). Treatment with lidocaine did not impact quality of life.

**DISCUSSION:**

Intravenous lidocaine in patients with painful PDAC and CP did not show an overall clinically relevant reduction of pain. However, this pilot study shows that the treatment is feasible in this patient group and had a positive effect in a third of patients which lasted up to a month with only minor side effects. To prove or exclude the efficacy of intravenous lidocaine, the study should be performed in a study with a greater sample size and less heterogeneous patient group.

## INTRODUCTION

Refractory pain is a major clinical problem for many patients with pancreatic ductal adenocarcinoma (PDAC) and chronic pancreatitis (CP). Nearly 75% of patients with PDAC suffer from pain at the time of diagnosis, increasing up to 90% of patients in advanced stages (1). Despite current multimodal pain management, 60% of patients with PDAC and pain remain undertreated (2). Similarly, pain is reported in 80%–90% of patients with CP, which strongly impairs quality of life (QOL), and is the leading cause of hospitalization, disability, and unemployment (3–5).

Initially, pain in these patients is addressed using the World Health Organization analgesic ladder (6), with the addition of antiepileptics and antidepressants (7,8). However, more advanced pain therapies are often necessary (9,10). These include celiac plexus or splanchnic nerve blocks with neurolytic agents and/or radiofrequency lesioning or intrathecal analgesia (10–12). The downside is that these therapies are invasive and have temporary effect, while CP is a chronic disease. At the same time, in patients with PDAC, local advancement and metastatic progression continue to activate pain nociceptors and inflammation (5). Moreover, for patients with CP and PDAC, opioids are frequently prescribed, leading to abuse, misuse, and tolerance (10,13).

Over the past decade, the clinical use of intravenous lidocaine gained increased attention. Lidocaine has inhibitory effects on ion channels and receptors, causing an anti-inflammatory, analgesic, and antihyperalgesic effect (14,15). Perioperative lidocaine infusion, improved rehabilitation, shortened hospital stay, and reduced chronic postsurgical pain in patients undergoing spine surgery (14,16,17). A systematic review including patients with different diseases causing neuropathic pain (e.g., due to stroke, spinal cord injury, cancer, and polyneuropathy) showed that intravenous lidocaine was more effective than placebo in reducing pain without causing adverse events (18). Also, a single intravenous infusion of lidocaine provided greater magnitude and duration of pain relief than a placebo in opioid refractory patients with pain from various distributions of cancer disease (i.e., upper extremity, lower extremity, head and neck region, chest, abdominal, and retroperitoneal) (19). These promising results of an established drug, with low patient burden, and straight-forward implementation in the clinic have led to a more frequent use in PDAC and CP within our experience in the Netherlands.

Some centers in the Netherlands are using intravenous lidocaine as part of pain treatment for patients with PDAC and CP. However, data from prospective multicenter studies into its efficacy, patient satisfaction, and adverse events are lacking. Therefore, this study aimed to determine the efficacy and safety of monitored lidocaine infusion in patients with painful PDAC and CP. We hypothesized that lidocaine is safe and leads to a clinically relevant reduction in the mean difference in pain severity on day 1.

## METHODS

### Study design

This is a multicenter nonrandomized prospective pilot study. For this trial, ethical approval was obtained at the Medical Ethical Committee of the Amsterdam UMC (January 14, 2021, W21_005 # 21.007), and this was confirmed and approved by the Ethical Boards of all participating centers. The trial was designed in accordance with the Consolidated Standard of Reporting Trials guidelines (20) and registered at ClinicalTrials.gov: NCT04048278. The funders of the study had no role in the study design, data collection, data analysis, data interpretation, writing of the report, and decision to publish.

### Patients

Patients were included between July 2021 and November 2022 in 5 Dutch hospitals: Amsterdam UMC (2 locations), Leiden University Medical Center, Maastricht University Medical Center+, and Rijnstate Hospital Arnhem. Patients were eligible for inclusion when diagnosed with PDAC (pathologically confirmed diagnosis, all stages, with a life expectancy ≥ 3 months) or CP (based on the M-ANNHEIM diagnostic criteria (21)); Numeric Rating Scale (NRS) score ≥4 despite previous pain treatment with nonopioid analgesics, opioids, tricyclic antidepressants, or a NRS <4, but unable to reduce opioids; aged 18 years or older; and Eastern Cooperative Oncology Group performance status 0–2. Exclusion criteria comprised patients with contraindications for intravenous lidocaine (i.e., inadequate liver function, hypersensitivity to local anesthetics, New York Heart Association Class III or IV, cardiac disease or myocardial infarction within the past 12 months, shock, and conduction abnormalities [defined as second-degree and third-degree atrioventricular blocks or atrial fibrillation]) and patients who underwent invasive pain therapies: endoscopic treatment or surgery, splanchnic nerves or celiac plexus block, neurolysis, or radiofrequency lesioning.

### Procedures

**Figure 1.**
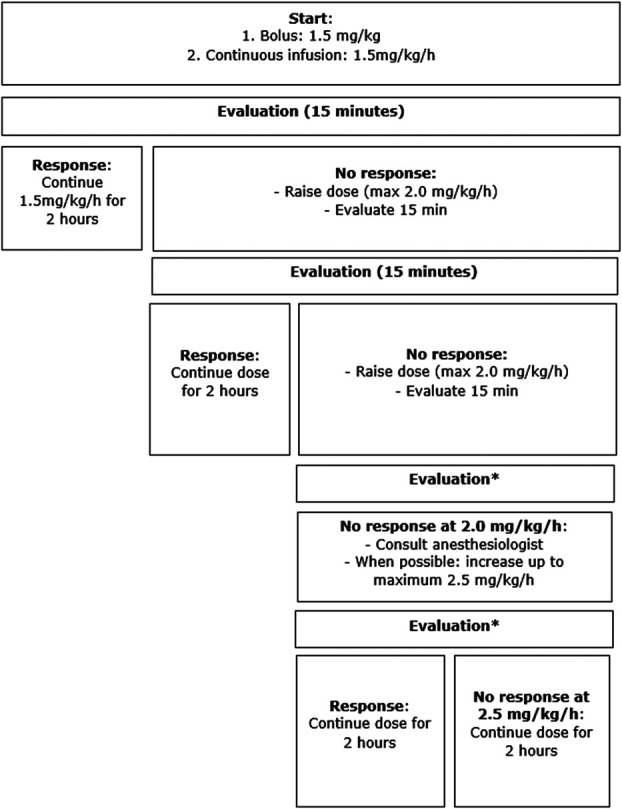
Lidocaine administration. *Evaluation is done every 15 minutes, but the increase in dose is decided by the treating clinician. Therefore, it can take more evaluations to get to 2.0 mg/hr, and exact times are not specified in this figure.

Monitored intravenous lidocaine infusion for patients with PDAC and CP was current practice in all participating centers. Patients underwent continuous monitoring during infusion with 3-lead electrocardiogram, SpO2, and blood pressure control every 15 minutes. Based on local protocols and availability of staff, patients were (short-term) clinically admitted for infusion at the postanesthesia care unit or underwent treatment at an ambulatory pain center within the hospitals outpatient clinic. First, a lidocaine bolus of 1.5 mg/kg was administered, followed by a continuous infusion of 1.5 mg/kg/hr (22). Pain scores were evaluated every 15 minutes. In case of a meaningful pain reduction (i.e. at least 2 points [or 30%] on the NRS scale from 0 to 10 from start infusion), the dose administered was continued for 2 hours. When no pain reduction was noticed, the dose was raised every 15 minutes up to a maximum of 2 mg/kg/hr. If at the maximum doses again no pain reduction was present, the anesthesiologist was consulted. In the absence of side effects and with permission of the anesthesiologist, lidocaine infusion was increased to a maximum of 2.5 mg/kg/hr or 250 mg/hr. This process is described in Figure 1. Following treatment, the patient was observed for 1 hour before discharge from the hospital. Responders could be scheduled for additional lidocaine treatment after 2 months.

### Data collection

Data on baseline characteristics (i.e., age, sex, body mass index, current use of pain medication, comorbidities, Eastern Cooperative Oncology Group performance status, CP etiology, and tumor stage), specifics on the lidocaine infusion (i.e., duration infusion, maximum dosage, and minor and major side effects), and follow-up (i.e., additional lidocaine infusion, invasive CP treatment [i.e., endoscopy or surgery], tumor targeted therapy, additional invasive pain therapy, and survival) were collected using Castor Electronic Data Capture (EDC), Amsterdam. Patient-reported outcomes were collected at baseline (i.e., before start lidocaine infusion), 1 day after infusion, at 2 weeks, and one-three-six months (Supplementary Table 1, Supplementary Digital Content 1, http://links.lww.com/CTG/B184).

Tumor stage was defined as resectable, borderline resectable, locally advanced, metastatic, or recurrent in case of PDAC (23). Current pain medication was described using the WHO analgesic ladder, as WHO 1 (nonopioid), 2 (mild-opioid), or 3 (strong opioid), and additional antiepileptics and antidepressants were noted (6). Potential side effects were included in the Castor case report form and a place to write free text; for minor side effects, these included vertigo, nausea, vomiting, hypotension metal taste, tinnitus, and tingling of mouth and lips. Possible major side effects included anaphylactic reaction, cardiac arrhythmias, and neurologic symptoms.

Patient-reported outcomes included the Brief Pain Inventory (BPI) (24,25), NRS (26), Izbicki pain score (27) (only for patients with CP), Global Perceived Effect (GPE) (28), and the Short-Form survey (SF-12) (29). The BPI evaluates the severity of pain on 5 domains and pain interference on 7 domains. The scores reported are the mean scores of the individual domains. Scores range on a scale from 0 to 10; a higher score indicates more pain. The NRS score ranges from 0 to 10, and the Izbicki pain score ranges from 0 to 100, in which higher scores indicate more pain. A clinically relevant decrease/response in pain was defined as Δ ≥ 1.3 (on a scale from 0 to 10) or Δ ≥ 13 (on a scale from 0 to 100) (30). The GPE scale comprises 2 domains: recovery of complaints and treatment satisfaction (ranging from 1 to 7). Higher scores indicate less effect of the treatment: a score of 1–2 was associated with clinically relevant improvement, 3–5 stable, and 6–7 clinically relevant deterioration (31). QOL physical (PCS) and mental score (MCS) were evaluated in which higher scores indicate improved QOL. A mean difference of 3 points in MCS/PCS was considered clinically relevant (32,33).

### Outcomes

The primary endpoint was the mean difference in pain severity between baseline and day 1 after the infusion evaluated by the BPI scale (0–10 points).

Secondary endpoints included the duration of infusion, dose of lidocaine administered, minor and major side effects, and number of lidocaine infusions performed. In addition, short-term and long-term effects of pain therapy (evaluated by the BPI, NRS, and Izbicki pain score, GPE) and QOL (evaluated by the SF-12) were evaluated. The effects of pain therapy included mean differences between baseline and the follow-up time points and proportion of patients with a relevant reduction in pain severity (responders).

### Sample size calculation

Since this is an exploratory study and very few data are available, no formal sample size calculation was performed. Initially, we aimed to include 30 patients of whom 15 patients with PDAC and 15 patients with CP. Owing to slow accrual within the CP arm, possibly new insights related to the efficacy of early surgery for CP (9), the sample size was adjusted to 30 patients regardless of diagnosis PDAC and CP. When patients withdrew informed consent or did not receive the intervention, they were replaced according to the protocol. Post hoc we did make a formal power calculation based on the primary outcome. A sample size of 30 patients will have 80% power to detect a difference in means of 1,3 (e.g., a first condition mean, μ₁, of 1 and a second condition mean, μ₂, of −0,3), assuming a standard deviation of differences of 2,456, using a paired *t*-test with a 5% 2-sided significance level.

### Statistical analysis

Descriptive statistics were used to give an overview of the baseline characteristics. Results were reported as proportions for categorical variables and as mean with SD or median with interquartile range for continuous variables. Normally distributed data were compared using a Students-*T*-test, categorical data using the χ^2^ test or Fisher exact test, and nonnormally distributed data using the Mann-Whitney *U* test.

To evaluate the effect of the intervention, mean differences (Δ) in pain and QOL scores between baseline and the different follow-up time points were calculated. In addition, the percentage of patients who reported a clinically relevant response was reported. Subgroup analyses were performed on patients with CP and PDAC and patients who had a clinical response within primary analysis (based on the BPI). Sensitivity analysis was performed using 30% pain relief (relative outcome measure) as clinically significant pain reduction evaluated by the BPI questionnaire (34). Missing data were reported but not imputed because of the missing data being not at random. A *P*-value of <0.05 was considered statistically significant. However, as this was only an exploratory study, with no formal sample size calculation, no comparison of treatments among groups was made and only clinical relevance was reported. Analyses were performed in RStudio version 4.2.1.

## RESULTS

**Figure 2.**
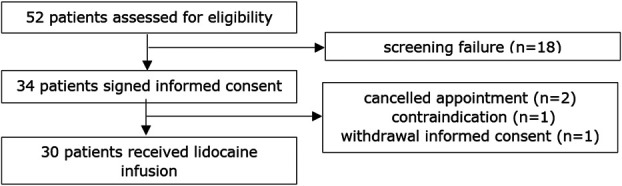
Trial profile.

Among the 52 patients eligible for inclusion, 33 patients signed informed consent (Figure 2). Four patients were excluded before intervention, due to cancellation of the appointment (n = 2), a contraindication at the day of the appointment (new atrial flutter, n = 1), and withdrawal of informed consent (n = 1). In total, 30 patients were included in the final cohort and received intravenous lidocaine (Table 1).

**Table 1.** Baseline characteristics

	Total	CP	PDAC
	n = 30	n = 11	n = 19
Age, median IQR	61.0 (54.0–67.0)	53.0 (50.5–61.0)	65.0 (57.0–70.0)
Female/male	13/17	2/9	11/8
BMI (median IQR)	22.0 (20.6–24.6)	22.4 (20.5–24.5)	21.9 (20.7–24.5)
Missing		1	1
Comorbidities^a^			
None	12 (40%)	2 (27%)	9 (47%)
One comorbidity	9 (30%)	4 (36%)	2 (26%)
Multimorbidity	9 (30%)	4 (35%)	2 (26%)
Pain medication (WHO ladder)			
1 (nonopioid)	6 (20%)	2 (18%)	4 (21%)
2 (mild-opioid)	3 (10%)	1 (9%)	2 (11%)
3 (strong-opioid)	21 (70%)	8 (73%)	13 (69%)
ECOG performance status^b^			
0	5 (26%)		5 (26%)
1	10 (53%)	NA	10 (53%)
≥2	4 (21%)		4 (21%)
Tumor stage PDAC^b^			
Locally advanced	9 (47%)		9 (47%)
Metastatic	8 (42%)	NA	8 (42%)
Recurrence	2 (11%)		2 (11%)
Etiology CP^c^			
Toxic metabolic	6 (55%)	6 (55%)	
Idiopathic	2 (18%)	2 (18%)	NA
Obstructive	1 (9%)	1 (9%)	
Other/unknown	2 (18%)	2 (18%)	

No missing values unless indicated otherwise.

BMI, body mass index; CP, chronic pancreatitis; ECOG, Eastern Cooperative Oncology Group; IQR, interquartile range; NA, not applicable; PDAC, pancreatic ductal adenocarcinoma; TCA, tricyclic antidepressants.

a Registered are cardiac, diabetes mellitus, vascular, pulmonary, neurologic/cerebrovascular, gastrointestinal/hepatic, urogenital, thrombotic/coagulopathy, connective tissue disease, AIDS/HIV, other malignancies.

b Only for patients with PDAC.

c Only for patients with CP.

Of these, 19 patients (63%) were diagnosed with PDAC and 11 patients (37%) with CP. Median age was 61 years (IQR: 54–67), 13 were female (43%) and 17 male (57%). Patients with PDAC had locally advanced disease in 9 of 19 (47%), metastatic disease in 8 of 19 (42%), and recurrent disease in 2 of 19 (11%). Patients with CP had an etiology of toxic metabolic in 6 of 11 (55%), idiopathic in 2 of 11 (18%), obstructive 1 of 11 (9%), and unknown in 2 of 11 (18%).

### Lidocaine infusion

After initial 1.5 mg/kg lidocaine bolus infusion, the median duration infusion was 120 minutes (IQR: 96–180), with a median dose of 150 mg/hr (Table 2). Minor side effects were seen in 3 patients (10%), nausea, tingling of mouth/lips, and vertigo. These side effects were short-term and stopped after the infusion. No major side effects were observed. In total, 8 of 30 patients (27%) received a second lidocaine infusion (Table 2).

**Table 2.** Lidocaine infusion

	First infusion n = 30	Second infusion n = 8
Duration infusion in min, median (IQR)	120 (96–180)	118 (93–133)
Maximum dosage (mg/hr), median (IQR)	150 (132–175)	147 (135–163)
Minor complications of which	3 (10%)	2 (25%)
Nausea	1 (3%)	0 (0%)
Tingling of mouth and lips	1 (3%)	0 (0%)
Vertigo	1 (3%)	0 (0%)
Metal taste	0 (0%)	1 (13%)
Other	0 (0%)	1 (13%)
Major complications	0 (0%)	0 (0%)

No missing values unless indicated otherwise.

IQR, interquartile range.

### Treatment response: day 1

Regarding the primary endpoint, patients reported a mean BPI severity reduction of 0.8 (SD ± 1.5), and this was lower than the predefined Δ ≥ 1.3 and not considered clinically relevant (Table 3). On day 1, 9 of 29 patients (31%) had a clinically relevant decrease in BPI severity (Δ ≥ 1.3) after lidocaine infusion (Table 3: 1 patient missing in BPI evaluation due to missing data). Two of 11 patients with CP (18%) were responders compared with 7 of 18 (39%) patients with PDAC.

**Table 3.** Short-term effect on pain (day 1)

	All patients (n = 30)				CP (n = 11)				PDAC (n = 19)			
	Baseline	1 d	Mean difference^a^	N patients response (%)	Baseline	1 d	Mean difference^a^	N patients response (%)	Baseline	1 d	Mean difference	N patients response (%)
Mean score BPI (SD)^b^												
Severity of pain 0-10 (mean)	5.9 (1.6)	4.9 (2.4)	−0.8 (1.5)	9/29 (31%)	5.8 (1.9)	4.6 (2.5)	−1.1 (1.3)	2/11 (18%)	6.1 (1.1)	5.6 (2.1)	−0.5 (1.7)	7/18 (39%)
Pain interference 0–10 (mean)	5.6 (2.4)	4.6 (2.5)	−0.9 (1.1)	10/28 (36%)	5.4 (2.5)	4.0 (2.8)	−1.2 (0.8)	2/11 (18%)	6.0 (2.2)	5.5 (2.0)	−0.5 (1.4)	8/17 (47%)
Numeric rating scale												
Average pain (0–10)	6.3 (1.7)	5.8 (1.9)	−0.5 (1.1)	5/30 (17%)	6.0 (1.9)	5.6 (2.1)	−0.4 (1.2)	2/11 (13%)	6.7 (1.3)	6.1 (1.4)	−0.6 (0.9)	3/19 (16%)
Worst pain (0–10)	8.1 (1.6)	7.5 (1.8)	−0.6 (0.9)	5/30 (17%)	7.9 (1.6)	7.4 (1.8)	−0.6 (0.9)	2/11 (13%)	8.3 (1.5)	7.7 (1.9)	−0.6 (0.9)	3/19 (16%)
Izbicki pain score (0–100)^c^	NA	NA	NA	NA	75.5 (17.4)	73.9 (12.1)	1.6 (12.6)	1/10 (10%)	NA	NA	NA	NA
Global perceived effect												
Recovery of complaints (1–7)	NA	3.4 (0.9)	NA	5/30 (17%)	NA	3.4 (0.9)	NA	2/11 (18%)	NA	4.0 (1.0)	NA	3/19 (16%)
Treatment satisfaction (1–7)		2.7 (1.4)		16/30 (53%)		2.9 (1.5)		7/11 (64%)		2.5 (1.2)		1/19 (47%)

No missing values unless indicated otherwise.

BPI, Brief Pain Inventory; CP, chronic pancreatitis; NA, not applicable; PDAC, pancreatic ductal adenocarcinoma.

a Clinically relevant response was defined as Δ < −1.3.

b One patient missing in BPI, severity of pain, and 2 patients in BPI, pain interference (both PDAC groups).

c One patient missing.

Treatment responses on the other questionnaires on day 1 were observed for 17% of patients for the NRS, 10% on the Izbicki pain score, 17% on GPE recovery of complaints, and 53% on treatment satisfaction. The GPE treatment satisfaction showed 16 of 30 (53%) (very) satisfied patients, 4 of 30 (13%) a little satisfied, 6 of 9 (20%) neutral, 3 of 30 (10%) a little unsatisfied, and 1 of 30 (3%) not satisfied. The GPE recovery of complaints (very) much improved in 5 of 30 patients (17%), with a little improvement in 9 of 30 (30%), no change in 15 of 30 (50%), and deterioration in 1 of 30 (3%).

### Treatment response: 2 weeks–6 months

**Figure 3.**
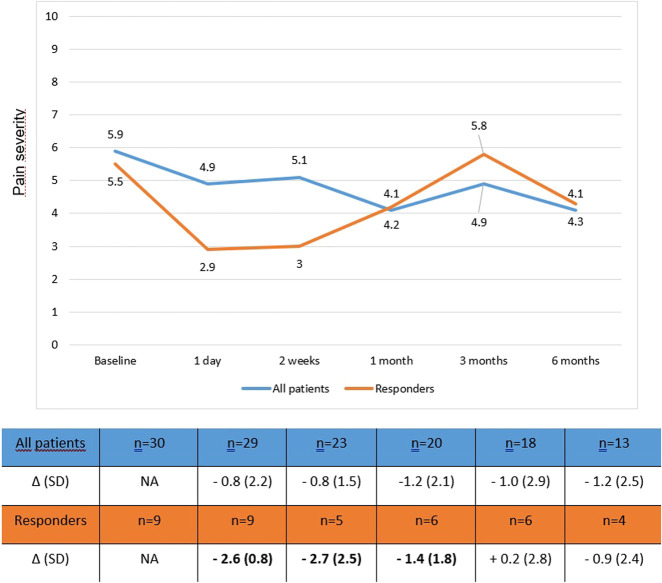
Long-term effect on pain after lidocaine infusion in responders versus all patients. Numbers in the graph indicate the mean BPI pain severity scores at the different time points. Responders are patients with a clinically relevant reduction in pain severity (defined as Δ ≥ 1.3). Bold numbers in the table indicate a clinically relevant difference.

Taking all patients into account, BPI severity improved over time in all patients, but did not reach the clinically relevant Δ −1.3 (range Δ: −0.8 to −1.2, Supplementary Table 2, Supplementary Digital Content 1, http://links.lww.com/CTG/B184). Treatment response in the 9 of 29 patients with a clinically relevant response, lasted up to 1 month after treatment (Figure 3, Supplementary Table 3, Supplementary Digital Content 1, http://links.lww.com/CTG/B184). In the subgroup of patient with CP, a clinically relevant mean difference BPI pain severity after 1, 3 months could be measured (Supplementary Table 2, Supplementary Digital Content 1, http://links.lww.com/CTG/B184).

The NRS pain scores gradually improved from an average of 6.3 (SD ± 1.7) to 4.1 (SD ± 2.9) after 6 months (range Δ: −1.5 to −3.0, Supplementary Table 2, Supplementary Digital Content 1, http://links.lww.com/CTG/B184). The GPE recovery of complaints ranged from 2.9 to 3.7 and treatment satisfaction from 2.9 to 3.8, with 4 being “no change/neutral middle” on the scale from 1 to 7 (Supplementary Table 2, Supplementary Digital Content 1, http://links.lww.com/CTG/B184). The Izbicki pain score showed no clinically relevant change, except at 3 months (Δ-20.9, SD ± 34.21).

### Pain medication and additional therapies

Most of the patients (12/21, 52%) reported after 2 weeks that their pain medication remained the same than before the infusion, 5 of 21 (24%) used less, and 6 of 21 (29%) used more pain medication (Table 4A). Over the following months, the percentage of patients who used less medication increased; however, patients underwent additional (minimal) invasive treatments (e.g., chemotherapy and plexus blocks) and data were missing. Overall, 16 of 30 patients (53%) underwent additional (minimal) invasive treatments over time (Table 4B). Patients with initial response (n = 9) underwent these invasive therapies equally to those without response (14% versus 14%, Supplementary Table 4, Supplementary Digital Content 1, http://links.lww.com/CTG/B184). Splanchnic/coeliac blocks were only performed in patients who had no initial response.

**Table 4.** (A). Use of pain medication during 6-mo follow-up. (B). Additional treatment during 6-mo follow-up

(A) All patients (n = 30)	2 wk	1 mo	3 mo	6 mo
Change in medication				
Less medication	5 (22%)	4 (20%)	6 (33%)	5 (40%)
No change	12 (52%)	10 (50%)	5 (28%)	4 (31%)
More medication	6 (26%)	6 (30%)	7 (39%)	4 (31%)
Missing	9	10	12	17

(B) Type of treatment^a^	All patients (n = 30)	Responders (n = 9)	Chronic pancreatitis (n = 11)	Pancreatic ductal adenocarcinoma (n = 19)
Endoscopy	1 (3%)	1 (11%)	1 (9%)	0
Surgery	3 (10%)	2 (22%)	2 (18%)	1 (5%)
Coeliac/splanchnic block	5 (17%)	0	1 (9%)	4 (21%)
Chemotherapy	11 (37%)	5 (56%)	0	11 (58%)
Radiotherapy/irreversible electroporation	2 (7%)	1 (11%)	0	2 (11%)
No additional treatment	14 (46%)	4 (44%)	7 (64%)	7 (37%)

a Some patients had multiple (minimal) invasive treatments, e.g., chemotherapy and surgery.

### QOL

In the overall group, the mean PCS ranged from 31.6 (SD ± 10.6) to 31.1 (SD ± 14.4) over the study period and no clinically relevant mean difference was found (Table 5). MCS ranged from 34.4 (SD ± 7.7) to 36.6 (SD ± 7.8), which was higher in the first month after lidocaine infusion with a mean difference of 3.5 (SD ± 12.3). In the subgroup of patients with CP, the PCS was higher at all the time points compared with baseline with mean differences ranging from Δ 4.2 to Δ 7.7 but did not reach clinical relevance of Δ ≥ 3. For the patients with PDAC, PCS did not change over time. The MCS was higher at 6 months, with 5 of 19 (26%) patients remaining to answer the questionnaire.

**Table 5.** SF-12 questionnaire

	Baseline	1 mo	Mean difference	3 mo	Mean difference	6 mo	Mean difference
	n = 30	n = 20		n = 18		n = 13	
PCS (SD)	31.6 (10.5)	35.8 (10.7)	+1.6 (8.8)	32.7 (13.9)	+0.6 (14.4)	31.1 (14.4)	0.7 (11.2)
MCS (SD)	34.6 (7.8)	36.6 (7.8)	**+3.5 (12.3)**	34.9 (7.0)	+0.3 (9.0)	34.4 (7.7)	−0.9 (7.6)
Chronic pancreatitis							
	n = 11	n = 9		n = 8		n = 8	
PCS (SD)	29.4 (9.1)	36.7 (12.3)	**+7.7 (12.9)**	34.7 (15.3)	**4.2 (13.9)**	34.7 (15.3)	**4.2 (13.9)**
MCS (SD)	35.8 (7.0)	37.0 (9.0)	+0.9 (10.1)	34.8 (7.2)	−2.8 (4.9)	34.8 (7.2)	−2.8 (4.9)
Pancreatic ductal adenocarcinoma							
	n = 11	n = 9		n = 8		n = 8	
PCS (SD)	32.9 (11.3)	35.0 (9.7)	+0.0 (11.2)	31.2 (13.4)	−2.2 (14.8)	33.4 (16.5)	−0.9 (9.4)
MCS (SD)	33.2 (8.2)	36.2 (7.1)	+2.1 (9.9)	35.1 (7.3)	2.8 (10.9)	35.1 (9.2)	**3.3 (8.3)**

Higher scores indicate improved condition.

Bold numbers indicate clinical relevance (Δ ≥ 3.0).

CP, chronic pancreatitis; MCS, mental score; PCS, physical score; PDAC, pancreatic ductal adenocarcinoma; SF-12, Short-Form survey.

### Sensitivity analysis

Using the relative instead of absolute mean difference in pain scores (evaluated by the BPI questionnaire), patients pain at day 1 decreased with 16%. This was not considered clinically relevant, and this remained the same at all time points (Supplementary Table 5, Supplementary Digital Content 1, http://links.lww.com/CTG/B184).

## DISCUSSION

This first prospective multicenter pilot study on IV lidocaine in patients with painful PDAC and CP found a clinically relevant response in 31% of patients on day 1, with prolonged effect up to 1 month after initial treatment. Intravenous lidocaine seems safe in these patients, with only 10% minor complications and no major complications.

The efficacy of intravenous (or subcutaneous) lidocaine was suggested by previous studies investigating different patient groups (35,36). A randomized placebo-controlled trial in 50 patients with opioid refractory cancer pain (diagnosis not specified; mostly patients with head and neck pain [28%] and abdominal pain [19%]) showed a mean pain reduction of 6.3 points after infusion of lidocaine (scale: 0–10) compared with a 2.3 decrease after placebo treatment (19). This randomized trial demonstrated higher response rates than this study but also included a slightly higher dose of lidocaine (2.0 mg/kg, compared with the start dose of 1.5 mg/kg with). Moreover, a meta-analysis from 2019 investigating the effect of lidocaine included 4 RCTs, 1 positive (n = 50), and 3 negative (n = 10 each) trials (35). Pooled results demonstrated that a higher dose (4–5 mg/kg) over 30–80 minutes provided significant benefit for lidocaine (50% pain reduction) compared with placebo in cancer pain (35). The initial dose within the present trial (1.5 mg/kg) can be considered safe because only 10% of patients within the present trial reported mild side effects. Therefore, it would seem feasible to increase the dose or continue the administration for a more extended period to enhance and sustain effect. Moreover, a “mandatory” repetition of the infusion could be considered because within this study, only 27% received a second infusion on request. Specifically, a randomized placebo controlled trial including 32 patients with neuropathic pain showed an improvement in pain especially after the third or fourth infusion (37). However, the increase in patient burden should be considered in this case.

In this study, patients with PDAC benefitted the most from intravenous lidocaine (39%) compared with 18% for CP. Since most patients with PDAC were treated in a palliative care setting, disease progression could have counteracted an even longer-lasting treatment effect. As intravenous lidocaine seems easy to implement with low patient burden, it might be an adequate bridge to minimal-invasive pain treatment measures like a semipermanent nerve block toward the end of life (38,39).

A potential reason for the lack of response in patients with CP could be that pain could exist for longer time, and higher doses of opioids have been previously prescribed which results in a more complex and difficult to treat pain. Also, a continuous use of alcohol or tobacco can lead to continued abdominal discomfort, potentially influencing the results (40). In some patients with CP, the treatment response seemed to increase over time. The latter could be due to the use of other invasive pain therapies during the study period or to the anti-inflammatory lidocaine effect that outlasts the immediate analgesic effect (41). Specifically, CP is a chronic inflammatory condition, and lidocaine decreases the release of these inflammatory mediators (42,43). Moreover, the recruitment rate of patients with CP was low. Most likely, for patients with an anatomical substrate for the disease, e.g., pancreatic duct obstruction caused by strictures or stone, early (endoscopic or surgical) intervention is the treatment of choice, giving a more permanent pain release (44). Intravenous administration lidocaine should probably only be attempted in the subgroup of patients with CP where endoscopic and surgical treatment is not an option or has failed to reduce pain significantly.

Remarkable in this study is the difference in pain intensity and patient satisfaction. Although only 31% of patients report a clinically relevant decrease in pain intensity, 53% of patients are (very) satisfied after the treatment. The paradox of patients in pain, yet satisfied with their pain management, has been previously reported (45,46). Within a cross-sectional study (n = 316) in patients with cancer, more than 75% of patients are (very) satisfied with pain management, despite almost half still reporting moderate to severe pain (46). Moreover, within the present trial, all patients were referred to a pain specialist before lidocaine administration. This could be potentially contributed to the patient satisfaction because their pain was appropriately acknowledged, current pain medication was assessed/optimized, and other therapies were offered when lidocaine failed.

On the other hand, pain intensity is a somewhat subjective measure and the clinically relevant difference could differ from person to person (47). For example, when patients experience less pain, their activity can improve (increasing the pain) and consequently pain intensity score does not improve. This illustrates the relevance for reporting both QOL and pain scores, especially for patients within the palliative setting. Furthermore, within the long-term outcomes, there was a clinically relevant mean difference assessing the NRS scores at all time points, whereas this was only evident at 1 time point in the BPI. In addition, within pain medicine, often responders and nonresponders are identified. Therefore, it has been suggested to provide separate “responder analysis” because the molecular mechanisms that underlie pain may vary among individuals over time and produce interindividual variation in pain perception and response (48). Altogether, this highlights the complexity of assessing chronic pain in clinical trials (49).

This study must be interpreted considering some limitations. First, this is a nonrandomized study with lack of a comparison group (placebo or standard treatment). Moreover, the study included a relatively small number of patients with 2 distinct types of pain. Therefore, it lacks power to correct for patient populations and other relevant confounders. Second, no evidence of decreased opioid consumption was established. Only 22%–40% used “less pain medication” over time, but it is unknown whether this includes the use of opioids. Third, 3 patients had a baseline average NRS of <4, finding a clinically relevant difference in pain is challenging in these patients. Fourth, the interpretation of long-term outcomes is unclear. This is due to missing data and patients who underwent additional treatments which might have influenced pain perception. Specifically, QOL assessment at 6 months was reported for only 43% of patients, especially in the PDAC group, this can be due to disease progression/end of life. Fifth, pain has no linear course, and the mean difference was dependent on the baseline pain score, which is just one moment in time. Sixth, pain is measured at single time points and no pain diaries are included, this might introduce bias. Nevertheless, this study has many strengths as well. This is the first multicenter prospective study in 5 different Dutch hospitals investigating the effect of intravenous lidocaine in patients with PDAC and CP. This study not only investigates pain relief directly after treatment, but includes patient-reported outcomes with a long-term follow-up of 6 months after treatment.

Intravenous lidocaine in patients with painful PDAC and CP did not show a clinically relevant reduction of the mean pain severity in all patients. However, this pilot study shows that the treatment is feasible in this patient group and had a positive effect in a third of patients which lasted up to a month, with only minor side effects. Therefore, to prove or exclude the efficacy of intravenous lidocaine, the study should be performed in a study with a greater sample size and less heterogeneous patient group. Up until then, lidocaine may be considered within a multimodal pain management strategy for patients in whom standard analgesic management is not sufficient, and endoscopic and surgical interventions are not feasible.

## CONFLICTS OF INTEREST

**Guarantor of the article:** Marc G. Besselink, MD, PhD.

**Specific author contributions:** S.A., M.B., and C.vV. were involved in conception and design of the study, acquisition of data, analysis and interpretation of data, drafting and revising critically for important intellectual content of all versions of the article, and gave final approval of this version of the manuscript to be published. M.B., B.B., S.B., M.B., O.B., W.tH., J.K., H.vK., M.N., N.S., M.S., R.V., J.dV., J.W., and J.vZ. were involved in conception and design of the study, interpretation of data, revising critically for important intellectual content of all versions of the article, and gave final approval of this version of the manuscript to be published. C.vE., M.B., and M.W. were involved in conception and design of the study, acquisition of data, interpretation of data, drafting and revising critically for important intellectual content of all versions of the article, and gave final approval of this version of the manuscript to be published.

**Financial support:** This research was funded by unrestricted grants from Inspire2Live, Deltaplan Alvleesklierkanker (grant number: WOO 22-01).

**Potential competing interests:** None to report.

**Data sharing statement:** Data can be made available on reasonable request by contacting the corresponding author.

**Study Highlights**

**WHAT IS KNOWN**

✓ Refractory pain is a major clinical problem in patients with pancreatic ductal adenocarcinoma (PDAC) and chronic pancreatitis (CP).
✓ New, effective therapies to reduce pain are urgently needed.
✓ Intravenous lidocaine is used in clinical practice in patients with PDAC and CP, but its efficacy has not been studied prospectively.

**WHAT IS NEW HERE**

✓ In 30 patients with PDAC and CP, treatment with intravenous lidocaine did not show a clinically relevant mean difference in pain severity 1 day after treatment.
✓ In one-third of patients (9/29, 31%), a clinically relevant response was observed which lasted up to 1 month.
✓ No serious complications were reported, only 3 minor complications (vertigo, nausea, and tingling of mount).

## Supplementary Material
