# Supplementary material for: Intravenous Lidocaine for Refractory Pain in Patients With Pancreatic Ductal Adenocarcinoma and Chronic Pancreatitis: A Multicenter Prospective Nonrandomized Pilot Study
Source: Clin Transl Gastroenterol. 2024 Aug 19;15(9):e1. doi: 10.14309/ctg.0000000000000760 (PMC11421722; doi:10.14309/ctg.0000000000000760)
Supplement: Supplementary file 1 [file ct9-15-e1j-s001.pdf]

Supplementary table 1. Time points of individual questionnaires

Questionnaire	Baseline	1 Day	1 week	1 month	3 months	6 months
Brief Pain Inventory	X	X	X	X	X	X
Numeric Rating Scale	X	X	X	X	X	X
Izbicki Pain score	X	X	X	X	X	X
Global Perceived Effect		X	X	X	X	X
Short Form Survey	X			X	X	X

Supplementary table 2. Long term pain scores

All patients	2 weeks	Mean difference	Response	1 month	Mean difference	Response	3 months	Mean difference	Response	6 months	Mean difference	Response
	n=23			*n=20*			*n=18*			*n=13*		
**Brief pain inventory**												
Severity of pain (0-10)	5.1 (2.6)	- 0.8 (2.2)	8/23 (35%)	4.1 (2.3)	- 1.2 (2.1)	8/20 (40%)	4.9 (2.7)	- 1.0 (2.9)	7/18 (39%)	4.3 (2.8)	- 1.2 (2.5)	5/13 (38%)
Pain interference (0-10)	4.8 (2.9)	- 0.9 (2.7)	8/23 (35%)	4.5 (2.8)	- 1.0 (2.7)	8/20 (40%)	4.1 (2.9)	**- 1.4 (2.9)**	11/18 (61%)	5.1 (3.5)	- 0.1 (2.7)	6/13 (46%)
**Numeric rating scale**												
Average pain (0-10)	5.2 (2.8)	**- 1.3 (2.4)**	7/23 (30%)	4.9 (2.5)	**- 1.3 (2.7)**	7/20 (35%)	4.6 (2.7)	**- 1.7 (2.8)**	9/18 (50%)	4.1 (2.9)	**- 2.1 (2.9)**	8/13 (62%)
Worst pain (0-10)	6.6 (2.9)	**- 1.5 (2.8)**	7/23 (30%)	6.1 (2.7)	**- 1.7 (2.8)**	8/20 (40%)	5.8 (2.9)	**- 2.1 (3.2)**	9/18 (50%)	6.2 (3.5)	**- 1.8 (3.3)**	4/13 (31%)
**Global perceived effect**												
Recovery of complaints (1-7)	3.9 (1.2)	NA	3/23 (13%)	3.9 (1.3)	NA	3/20 (15%)	2.9 (1.6)	NA	9/18 (50%)	3.2 (1.5)	NA	5/13 (38%)
Treatment satisfaction (1-7)	4.0 (1.9)		5/23 (22%)	3.2 (1.3)		5/20 (25%)	3.4 (1.9)		6/18 (33%)	3.0 (1.2)		4/13 (31%)
**Chronic Pancreatitis**	*n=10*			*n=9*			*n=8*			*n=8*		
**Brief pain inventory**												
Severity of pain (0-10)	5.3 (2.5)	- 0.9 (2.4)	4/10 (40%)	3.6 (2.5)	**- 2.3 (2.2)**	6/9 (67%)	4.1 (3.1)	**- 1.9 (3.2)**	4/8 (50%)	5.2 (2.5)	- 0.8 (2.3)	3/8 (38%)
Pain interference (0-10)	5.1 (3.2)	- 1.1 (2.7)	2/10 (20%)	4.4 (3.5)	**- 1.7 (3.7)**	5/9 (56%)	3.9 (1.3)	**- 1.9 (2.8)**	5/8 (63%)	6.1 (3.3)	+0.2 (2.3)	3/8 (38%)
**Numeric rating scale**												
Average pain (0-10)	5.5 (2.9)	**- 1.5 (2.6)**	4/10 (40%)	4.1 (2.5)	**- 2.6 (3.0)**	6/9 (67%)	4.1 (3.0)	**- 2.6 (3.9)**	5/8 (63%)	4.7 (2.9)	**- 2.2 (2.5)**	5/8 (63%)
Worst pain (0-10)	6.5 (3.3)	**- 2.2 (3.3)**	4/10 (40%)	5.2 (3.1)	**- 3.2 (3.2)**	5/9 (56%)	4.4 (3.1)	**- 2.6 (3.9)**	4/8 (50%)	7.4 (2.7)	**- 3.0 (4.0)**	2/8 (25%)
**Izbicki pain score (0-100)***	72.1 (21.1)	- 3.5 (27.4)	2/10 (20%)	63.2 (21.6)	- 10.9 (16.9)	2/8 (25%)	55.5 (33.1)	**- 20.9 (34.2)**	2/6 (33%)	69.9 (21.3)	- 4.8 (14.3)	1/7 (14%)
**Global perceived effect**												
Recovery of complaints (1-7)	3.7 (1.4)	NA	2/10 (20%)	3.1 (1.1)	NA	2/9 (22%)	2.8 (1.4)	NA	4/8 (50%)	3.1 (1.6)	NA	4/8 (50%)
Treatment satisfaction (1-7)	3.8 (1.9)		2/10 (20%)	2.9 (1.4)		3/9 (33%)	3.8 (2.1)		3/8 (38%)	2.9 (1.2)		3/8 (38%)
**Pancreatic Ductal Adenocarc**	*n=13*			*n=11*			*n=10*			*n=5*		
**Brief pain inventory**												
Severity of pain (0-10)	5.0 (2.7)	- 0.7 (2.2)	4/13 (31%)	4.9 (1.9)	- 0.4 (1.6)	2/11 (18%)	5.5 (2.4)	- 0.4 (2.8)	3/10 (30%)	3.1 (3.1)	**- 1.8 (2.9)**	2/5 (40%)
Pain interference (0-10)	4.5 (2.9)	- 0.8 (2.7)	6/13 (46%)	4.5 (2.3)	- 0.5 (1.5)	3/11 927%	4.3 (3.0)	- 0.9 (3.0)	6/10 (60%)	3.6 (3.6)	- 1.2 (3.8)	3/5 (60%)
**Numeric rating scale**												
Average pain (0-10)	4.9 (2.8)	- 1.1 (2.3)	3/13 (23%)	5.5 (2.4)	- 0.2 (1.9)	1/11 (0%)	5.0 (2.4)	**-1.6 (2.6)**	4/10 (40%)	3.2 (3.3)	**- 2.2 (2.5)**	3/5 (60%)
Worst pain (0-10)	6.6 (2.8)	- 1.0 (2.3)	3/13 (23%)	6.8 (2.2)	- 0.5 (1.9)	3/11 (27%)	6.1 (2.9)	**- 2.2 (3.5)**	5/10 (50%)	4.4 (4.2)	**- 3.0 (4.0)**	2/5 (40%)
**Global perceived effect**												
Recovery of complaints (1-7)	3.7 (1.4)	NA	3/13 (23%)	3.1 (1.1)	NA	1/11 (9%)	2.8 (1.4)	NA	5/10 (50%)	3.1 (1.6)	NA	1/5 (20%)
Treatment satisfaction (1-7)	3.8 (1.9)		3/13 (23%)	2.9 (1.4)		2/11 (18%)	3.8 (2.1)		3/10 (30%	2.9 (1.2)		1/5 (20%)

No missing values unless indicated otherwise. Higher scores indicate worse condition. Bold numbers indicate clinical relevant difference. **NA**: not applicable. *Only in n=10 at 2 weeks, n=8 at 1 month, n=6 at 3 months, and n=7 at 6 months.

Supplementary table 3. Long term pain scores in responders to treatment

	Baseline	1 day	Mean difference	N patients response	2 weeks	Mean difference	N patients response	1 month	Mean difference	N patients response
	n=9				*n=5*			*n=6*		
**Brief pain inventory**										
Severity of pain (0-10)	5.5 (1.3)	2.9 (1.8)	**- 2.6 (0.8)**	9/9 (100%)	3.0 (2.8)	**- 2.7 (2.5)**	4/5 (80%)	4.2 (1.9)	- **1.4 (1.8)**	3/6 (50%)
Pain interference (0-10)	4.3 (2.2)	2.9 (2.3)	**- 1.3 (0.9)**	6/9 (67%)	3.0 (2.3)	**- 1.6 (2.7)**	3/5 (60%)	5.1 (3.3)	+ 1.0 (2.6)	1/5 (20%)
**Numeric rating scale**										
Average pain (0-10)	5.3 (1.6)	4.7 (1.9)	- 0.7 (1.5)	2/9 (22%)	3.2 (2.9)	**- 2.4 (3.1)**	3/5 (60%)	5.0 (2.2)	- 0.7 (2.3)	1/6 (17%)
Worst pain (0-10)	7.6 (1.7)	6.3 (1.9)	- 1.2 (0.9)	3/9 (33%)	4.4 (2.5)	**- 3.0 (3.1)**	3/5 (60%)	5.8 (2.3)	**-1.3 (2.2)**	3/6 (50%)
**Global perceived effect**										
Recovery of complaints (1-7)	NA	2.7 (1.0)	NA	4/9 (44%)	3.6 (1.5)	NA	1/5 (20%)	3.8 (1.5)	NA	1/6 (17%)
Treatment satisfaction (1-7)		2.6 (1.2)		5/9 (56%)	3.4 (1.7)		2/5 (40%)	2.7 (1.0)		2/6 (33%)

	3 months	Mean difference	N patients response	6 months	Mean difference	N patients response
		n=6			*n=4*	
**Brief pain inventory**						
Severity of pain (0-10)	5.8 (2.3)	+ 0.2 (2.8)	1/6 (17%)	4.1 (2.9)	- 0.9 (2.4)	1/4 (25%)
Pain interference (0-10)	4.4 (2.8)	+ 0.5 (3.2)	2/6 (33%)	5.0 (3.4)	+ 1.9 (3.5)	1/4 (25%)
**Numeric rating scale**						
Average pain (0-10)	5.7 (1.6)	- 0.3 (2.2)	1/6 (17%)	4.3 (3.1)	- 0.5 (1.7)	1/4 (25%)
Worst pain (0-10)	6.7 (1.9)	**-1.3 (3.3)**	3/6 (50%)	5.5 (3.9)	- 1.0 (2.8)	1/4 (25%)
**Global perceived effect**						
Recovery of complaints (1-7)	3.0 (1.2)	NA	4/6 (67%)	4.0 (1.6)	NA	1/4 (25%)
Treatment satisfaction (1-7)	3.0 (1.8)		3/6 (50%)	3.0 (0.8)		1/4 (25%)

Supplementary table 4. Association of invasive treatments and change in medication and response

	2 weeks (n =21)		1 month (n=20)		3 months (n=18)		6 months (n=13)	
	No	Yes	No	Yes	No	Yes	No	Yes
**Less medication**	2 (17%)	2 (20%)	2 (18%)	2 (25%)	2 (18%)	4 (67%)	3 (43%)	2 (33%)
**No change**	6 (50%)	6 (60%)	7 (64%)	3 (38%)	3 (27%)	2 (33%)	2 (29%)	2 (33%)
**More medication**	4 (33%)	2 (20%)	2 (18%)	3 (38%)	6 (55%)	0 (0%)	2 (29%)	2 (33%)

	No invasive treatment	Invasive treatment
**No response**	10 (36%)	10 (36%)
**Response**	4 (14%)	4 (14%)

Supplementary table 5. Absolute versus relative difference in pain

BPI	Day one (n=30)					
	Baseline (mean, SD)	1 day (mean, SD)	Mean absolute difference^	Mean relative difference^	N patients response AD (%)	N patients response RD (%)
Severity of pain 0-10<br>Pain interference 0-10	5.9 (1.6)<br>5.6 (2.4)	4.9 (2.4)<br>4.6 (2.5)	- 0.8 (1.5)<br>- 0.9 (1.1)	 -16.1%<br>-21.6%	9/29 (31%)<br>10/28 (36%)	8/29 (28%)<br>8/28 (29%)
BPI	Two weeks (n=23)					
	Baseline (mean, SD)	Two weeks (mean, SD)	Mean absolute difference^	Mean relative difference^	N patients response AD (%)	N patients response RD (%)
Severity of pain 0-10<br>Pain interference 0-10	5.9 (1.6)<br>5.6 (2.4)	5.1 (2.6)<br>4.8 (2.9)	- 0.8 (2.2)<br>- 0.9 (2.2)	 - 11.2%<br>- 12.9%	8/23 (35%)<br>8/23 (35%)	7/23 (30%)<br>8/23 (40%)
BPI	One month (n=20)					
	Baseline (mean, SD)	One month (mean, SD)	Mean absolute difference^	Mean relative difference^	N patients response AD (%)	N patients response RD (%)
Severity of pain 0-10<br>Pain interference 0-10	5.9 (1.6)<br>5.6 (2.4)	4.1 (2.3)<br>4.5 (2.8)	- 1.2 (2.1)<br>- 1.0 (2.7)	 - 18.5%<br>- 12.9%	8/20 (40%)<br>8/20 (40%)	7/20 (35%)<br>8/20 (40%)

BPI	Three months (n=18)					
	**Baseline (mean, SD)**	**Three months (mean, SD)**	**Mean absolute difference^**	**Mean relative difference^**	**N patients response AD (%)**	**N patients response RD (%)**
Severity of pain 0-10	5.9 (1.6)	4.9 (2.7)	-1.0 (2.9)	- 9.6%	7/18 (39%)	6/18 (33%)
Pain interference 0-10	5.6 (2.4)	4.1 (2.9)	**-1.4 (2.9)**	- 15.4%	11/18 (61%)	9/18 (50%)
**BPI**	**Six months (n=13)**					
	**Baseline (mean, SD)**	**Six months (mean, SD)**	**Mean absolute difference^**	**Mean relative difference^**	**N patients response AD (%)**	**N patients response RD (%)**
Severity of pain 0-10	5.9 (1.6)	4.3 (2.8)	-1.2 (2.5)	- 19.7%	5/13 (39%)	5/13 (39%)
Pain interference 0-10	5.6 (2.4)	5.1 (3.5)	-0.1 (2.7)	+10%	6/13 (46%)	5/13 (39%)

BPI: brief pain inventory, **AD**: absolute difference, **RD**: relative difference
